# An interactive resource of molecular signalling in the developing human haematopoietic stem cell niche

**DOI:** 10.1242/dev.201972

**Published:** 2023-12-06

**Authors:** Edie I. Crosse, Anahi Binagui-Casas, Sabrina Gordon-Keylock, Stanislav Rybtsov, Sara Tamagno, Didrik Olofsson, Richard A. Anderson, Alexander Medvinsky

**Affiliations:** ^1^ Centre for Regenerative Medicine, University of Edinburgh, Edinburgh EH16 4UU, UK; ^2^ Omiqa Bioinformatics GmbH, Altensteinstraße 40, 14195 Berlin, Germany; ^3^ MRC Centre for Reproductive Health, University of Edinburgh, Edinburgh EH16 4TJ, UK

**Keywords:** Haematopoiesis, Human development, Spatial transcriptomics, Single cell RNA-seq, AGM region

## Abstract

The emergence of definitive human haematopoietic stem cells (HSCs) from Carnegie Stage (CS) 14 to CS17 in the aorta-gonad-mesonephros (AGM) region is a tightly regulated process. Previously, we conducted spatial transcriptomic analysis of the human AGM region at the end of this period (CS16/CS17) and identified secreted factors involved in HSC development. Here, we extend our analysis to investigate the progression of dorso-ventral polarised signalling around the dorsal aorta over the entire period of HSC emergence. Our results reveal a dramatic increase in ventral signalling complexity from the CS13-CS14 transition, coinciding with the first appearance of definitive HSCs. We further observe stage-specific changes in signalling up to CS17, which may underpin the step-wise maturation of HSCs described in the mouse model. The data-rich resource is also presented in an online interface enabling *in silico* analysis of molecular interactions between spatially defined domains of the AGM region. This resource will be of particular interest for researchers studying mechanisms underlying human HSC development as well as those developing *in vitro* methods for the generation of clinically relevant HSCs from pluripotent stem cells.

## Teaser

**Summary:** An in-depth and interactive exploration of molecular signalling in the human developmental haematopoietic stem cell niche at the spatial and single cell level.

## INTRODUCTION

Haematopoietic stem cells (HSCs) derive from the endothelium of the dorsal aorta (Ao) through the process of endothelial-to-haematopoietic transition (EHT), which has been extensively reviewed (Ottersbach, 2019; Wu and Hirschi, 2021). HSCs and intra-aortic haematopoietic clusters (IAHCs) predominantly emerge in the ventral domain of the Ao (AoV) (Tavian et al., 1996, 1999; Taoudi and Medvinsky, 2007; Jaffredo et al., 2013; Ivanovs et al., 2014). It is well documented that expression of some key molecules involved in haematopoietic development are ventrally polarised (Marshall et al., 2000; Peeters et al., 2009; Wilkinson et al., 2009) and experiments on the mouse aorta-gonad-mesonephros (AGM) region revealed an interplay of dorso-ventral signalling in HSC development (Souilhol et al., 2016a). The advent of spatial transcriptomics unveiled considerable dorso-ventral asymmetry in the AGM region and facilitated identification of markers and developmental regulators of HSCs (McGarvey et al., 2017; Crosse et al., 2020; Yvernogeau et al., 2020; Calvanese et al., 2022).

In the human embryo, the first definitive HSCs, which become transplantable, emerge during a limited time window between Carnegie Stage (CS) 14 and CS17 (33-41 days post fertilisation) in the AGM region (Ivanovs et al., 2011). Our previous studies of the signalling landscape of the human AGM region used laser capture microscopy (LCM) for targeted excision of areas of interest, focussing on CS16/CS17, the end of HSC generation in the human AGM region (Crosse et al., 2020). Here, we aimed to produce a more comprehensive picture covering the entire time window beginning from CS13, the stage immediately preceding the appearance of definitive HSCs. We generated a spatial transcriptomics resource augmented with an online tool for easy interactive exploration of molecular signalling in the AGM niche at CS13-CS16/CS17. The resource, available at https://medvinsky-lab.github.io/hsc-niche-explorer/, allows users to explore predicted niche ligand-receptor interactions across dorso-ventrally defined spatial domains and/or specific sub-populations defined by single cell RNA-seq. Our analysis reveals significant dynamic changes in signalling over the CS13-CS17 period, potentially underpinning gradual maturation of HSCs and progenitors in the human AGM region.

This study provides a valuable resource for researchers investigating the mechanisms underlying human HSC development, and for those developing *in vitro* methods for generating clinically relevant HSCs from pluripotent stem cells. By identifying stage-specific changes in molecular signalling during HSC development, this resource could aid in the development of more precise *in vitro* models for studying human haematopoiesis.

## RESULTS

### CS13-CS14 as a key checkpoint in HSC generation

#### Ventral polarisation of signalling at the appearance of definitive HSCs (CS13/CS14)

**Fig. 1.**
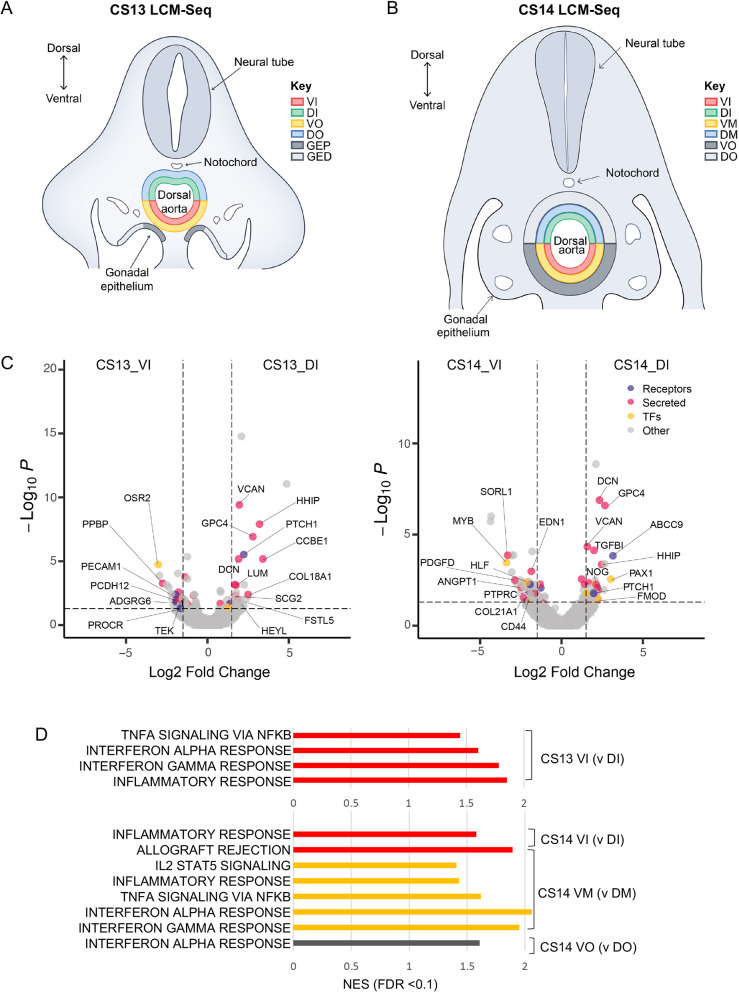
**Signalling polarisation across the dorsal-ventral axis of the early HSC developmental niche.** (A,B) Laser capture microdissection strategy for CS13 (*n*=2) and CS14 (*n*=2), respectively (DI, dorsal inner; DM, dorsal mid; DO, dorsal outer; GED, gonadal epithelium distal; GEP, gonadal epithelium proximal; VI, ventral inner; VM, ventral mid; VO, ventral outer). (C) VI versus DI differentially expressed genes for CS13 (left) and CS14 (right) (*P*.adj<0.05). (D) Normalised enrichment score for inflammatory pathways from differentially expressed genes for the comparisons indicated (FDR<0.1).

We investigated the spatial molecular landscape of the AGM region at CS13, immediately before HSC generation, and at CS14, corresponding to the onset of definitive HSC generation (Ivanovs et al., 2011). We took transverse 10 µm sections from the mid trunk of CS13 and CS14 embryos (*n*=2 embryos for each stage) using anatomical landmarks as guidelines to ensure the same region was taken across embryos. Sections were taken between the caudal appearance of the midgut loop and the caudal appearance of the liver and duodenum, before the bifurcation of the Ao (Fig. S1Ai,Aii). Within this region, the Ao contains the most and largest IAHCs (Tavian et al., 1996, 1999). For CS13, with LCM we sub-dissected concentric dorsal and ventral domains around the Ao (Fig. 1A). These domains included: (1) ventral and dorsal ‘inner’ domains (VI and DI, respectively) comprising the Ao luminal layer (endothelial lining and adjacent mesenchymal cells) and (2) ventral and dorsal ‘outer’ domains (VO and DO, respectively) comprising sub-aortic mesenchyme reaching dorsally to the notochord and ventrally to the gonadal epithelium. At this early stage, the sub-aortic mesenchyme represents a thin layer, so the Ao endothelial floor is in close vicinity to the gonadal epithelium outlining the urogenital ridges. Therefore, owing to the potential impact on IAHC/HSC development, we also sub-dissected (3) gonadal-epithelium into the proximal domain (GEP) adjacent to the ventro-lateral wall of Ao and the distal domain (GED), diverging from the Ao and descending ventrally (Fig. 1A). By CS14, the gonads become significantly distanced from the Ao floor due to thickening of the sub-aortic mesenchyme. Therefore, for CS14, urogenital ridges were excluded from analysis and the following six domains were sub-dissected: (1) VI and DI, (2) ventral and dorsal ‘mid’ domains (VM and DM, respectively) and (3) VO and DO, which comprise the Ao endothelial lining, sub-aortic mesenchyme/stroma and more distant outer mesenchyme/stroma, respectively (Fig. 1B). Transcriptome libraries from each of these spatial domains were generated and RNA-seq was performed. Concurrently, for both CS13 and CS14, we used consecutive sections immunostained for CDH5 (VE-cadherin, CD144) and RUNX1 (runt-related transcription factor 1) to validate the presence of ventrally localised CDH5^+^RUNX1^+^ IAHCs (Fig. S1B).

To determine dorso-ventral polarised signalling during the CS13-CS14 transition, we compared VI with DI and VO with DO for CS13, and VI with DI, VM with DM and VO with DO for CS14 (for full lists of genes see Table S1). From the differentially expressed genes (*P*adj<0.05) we identified secreted factors (Uniprot: KW-0964), receptors (GO: 0038023) and transcription factors (GO: 0003700). We found a notable consistency in genes upregulated dorsally in the DI at CS13 and CS14, including secreted molecules *VCAN*, *HHIP*, *DCN*, *GPC4* and receptor *PTCH1* (Fig. 1C). By contrast, ventral signalling (in VI) differed substantially between the two stages. Specifically, at CS14, VI was enriched for expression of genes relevant to EHT/HSC specification (VI versus DI) including transcription factors *MYB* (Mucenski et al., 1991; Oh and Reddy, 1999; Sakamoto et al., 2006) and *HLF* (Yokomizo et al., 2019; Calvanese et al., 2022); secreted factor *EDN1* (Crosse et al., 2020) and cell surface protein *CD44* (Oatley et al., 2020; Zhu et al., 2020), supporting the idea that CS14 is a key checkpoint in the HSC generation. Notably, for all pairwise comparisons of ventral and respective dorsal domains at CS13 and CS14, gene enrichment analysis (GSEA) showed ventralised enhancement for proinflammatory pathways (Table S1; Fig. 1D). These included ‘TNFA signalling via NfκB’ (CS13_VI, CS14_VM), ‘Interferon alpha/gamma response’ (CS13_VI, CS14_VM and CS14_VO) and ‘IL2-STAT5 signalling’ (CS14_VM). Ventral enrichment of ‘TNFA signalling via NfκB’ was previously reported at CS16 (Crosse et al., 2020).

#### Emergence of definitive HSCs is accompanied by sharp increase in complexity of ventralised signalling

**Fig. 2.**
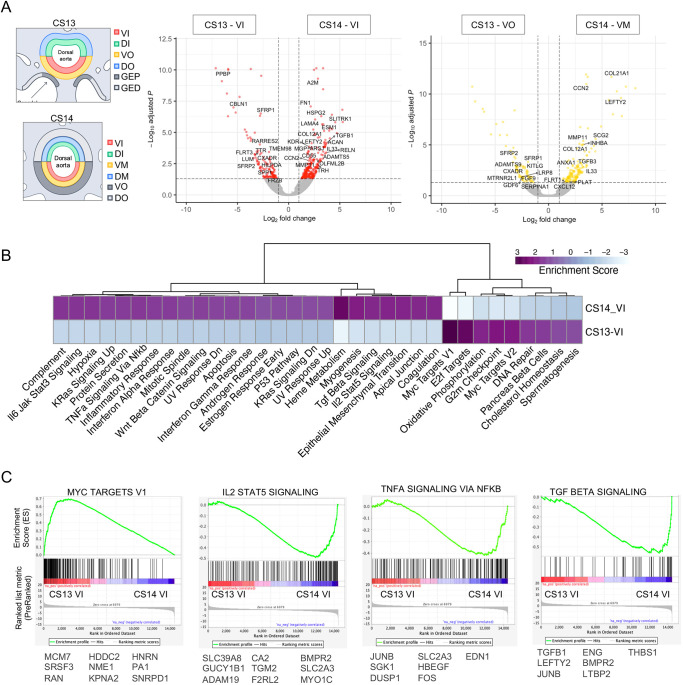
**Signalling pathway transitions in the HSC developmental niche from CS13 to CS14.** (A) Differentially expressed genes between the CS13 VI (*n*=2) and CS14 VI (*n*=2) (left) and between the CS13 VO (*n*=2) and CS14 VM (*n*=2) (right), with secreted factors highlighted (*P*.adj<0.05). For transcription factors see Fig. S2A. DI, dorsal inner; DM, dorsal mid; DO, dorsal outer; GED, gonadal epithelium distal; GEP, gonadal epithelium proximal; VI, ventral inner; VM, ventral mid; VO, ventral outer. (B) Normalised enrichment score for pathways from differentially expressed genes for CS13 VI versus CS14 VI (FDR<0.1). (C) Selected enrichment plots. Contributing genes (significantly differentially expressed, *P*.adj<0.05) are shown below each plot.

For deeper analysis of changes in the molecular milieu in AoV during the CS13-CS14 transition, we focused on the ventral luminal lining where IAHC/HSCs emerge: CS14_VI versus CS13_VI and deeper stromal layers, and CS14_VM versus CS13_VO (these domains are roughly equivalent in terms of size and distance from the Ao lumen) (Fig. 2A). We identified significant changes in expression of secreted factors, such as downregulation of pro-platelet basic protein (*PPBP*; CS14_VI versus CS13_VI) and WNT antagonists, secreted frizzled related proteins *SFRP1* and *SFRP2* (CS14_VI versus CS13_VI and CS14_VM versus CS13_VO) and frizzled related protein *FRZB* (CS14_VI versus CS13_VI). We also detected upregulation of TGF beta pathway factors: inhibin subunit beta A (*INHBA*; CS14_VM versus CS13_VO), *TGFB1*, *TGFB3* (CS14_VI versus CS13_VI) and left-right determination factor 2, (*LEFTY2*; CS14_VI versus CS13_VI and CS14_VM versus CS13_VO) (Fig. 2A). GSEA identified attenuation of cell-cycle-related pathways such as G2M checkpoint, E2F targets and MYC targets V1/V2 in CS14_VI (Fig. 2B,C). This decrease in cell proliferation can be explained by gradual endothelial maturation, which may mask the potential increase in proliferation of the smaller fraction of maturing haematopoietic stem and progenitor cells (HSPCs) (Batsivari et al., 2017; Canu et al., 2020).

A major finding of GSEA was a dramatic increase of signalling complexity in VI during the CS13-CS14 transition, coinciding with the onset of the generation of definitive HSC, which is reflected in the steep increase in the number of enriched pathways (Fig. 2B). It involves, among others, ventral intensification of proinflammatory pathways: ‘IL2 STAT5 signalling’, ‘IL6 JAK STAT3 signalling’, ‘TNFA signalling via NfκB’ and ‘Interferon alpha/gamma response’ (Fig. 2B,C). ‘TGF beta signalling’ also increased strongly in CS14_VI compared with CS13_VI, with significantly upregulated contributing genes *THBS1* (thrombospondin 1), *LTBP2* (latent transforming growth factor beta binding protein 2), *BMPR2* (bone morphogenetic protein receptor type 2), *ENG* (endoglin) and *JUNB* (JunB proto-oncogene), in addition to previously mentioned *INHBA*, *TGFB1*, *TGFB3* and *LEFTY2* (Fig. 2B,C).

We also detected a strong activation of the epithelial-mesenchymal transition (EMT) pathway in VI during the CS13-CS14 transition (Fig. 2B; Fig. S2B). Given the mechanical similarities between EMT and EHT, upregulation of EMT genes could contribute to HSC development (Ottersbach, 2019). We also observed an increase in the hypoxia pathway during this transition, consistent with previous reports implicating hypoxia in EHT (Imanirad et al., 2014) (Fig. 2B). These changes, which were also observed in the deeper ventral subendothelial layers, suggest a qualitative change in the HSC niche and consolidation of signalling through AoV layers, as evidenced by the shared pathways between the luminal and underlying stromal layers (Fig. S2C). Indeed, six out of nine ventrally enriched pathways are shared by both CS13_VI and CS13_VO and 16 out of 26 ventrally enriched pathways are shared by both CS14_VI and CS14_VM (Fig. 2; Fig. S2C; Table S1). Full GSEA pathway analysis for both CS14_VI versus CS13_VI and CS14_VM versus CS13_VO pairwise comparisons can be found in Table S1. Our findings suggest complex and dynamic endothelial-stromal interactions involved in the regulation of HSC development, which are explored in detail in the section ‘Characterisation of haematoendothelial and stromal populations’.

### Pathway dynamics in AoV during CS13-CS17

#### Hallmark pathway patterns

**Fig. 3.**
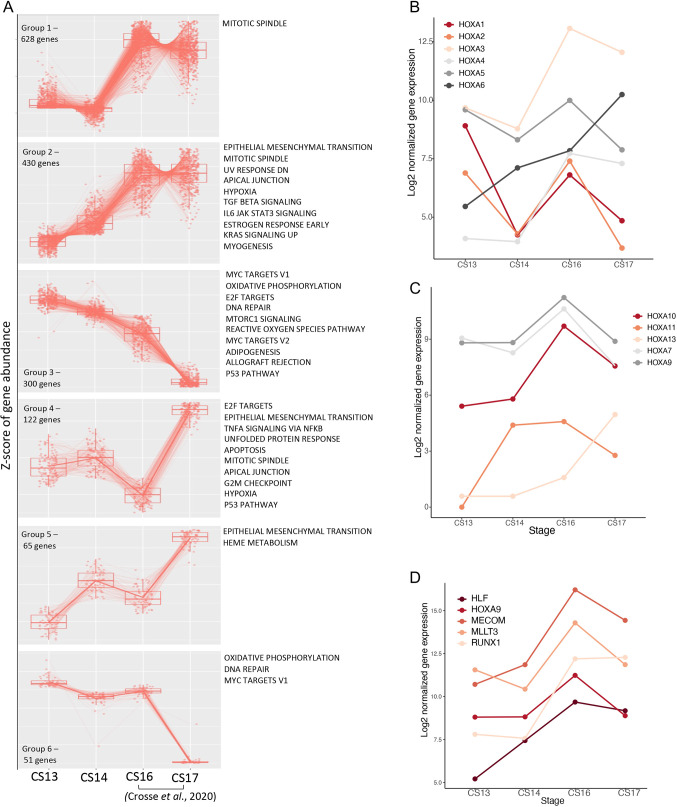
**Signalling pathway dynamics across the HSC developmental window.** (A) Dynamics of gene expression from CS13-CS17 showing the top six patterns of expression change and pathway enrichment for each pattern (FDR<0.1). (B-D) Dynamics of normalised gene expression of HOXA transcription factors (B-C) and developing HSC transcription factors (D) across CS13-CS17.

To gain insight into underlying signalling dynamics over the CS13-CS17 time window, we focused on the Ao inner luminal layer (VI) where EHT takes place. To this end, we combined our current CS13_VI and CS14_VI LCM-seq datasets described above with CS16_VI and CS17_VI LCM-seq datasets published previously (Crosse et al., 2020). We identified six major dynamic patterns of gene expression in the VI across the CS13-CS17 time window (Fig. 3A). Of particular interest was Group 2, comprising a broad array of genes with monotonically rising expression, which correlates with IAHC/HSC production across this period. Within this group, ‘TGF beta signalling’, ‘IL6 JAK STAT3 signalling’, ‘EMT’ and ‘Hypoxia’ pathways increase up to CS16/17. Conversely, Group 3 genes show monotonic decrease of expression from CS13 to CS17, including pathways identified above during the AoV CS13-CS14 transition, such as ‘Oxidative phosphorylation’. Progressive downregulation of cell-proliferation-related pathways ‘E2F targets’ and ‘Myc targets V1/V2’ may be associated with gradual cell maturation of endothelial cells (Figs 2B and 3). Of interest, during CS16-CS17 transition, Group 2 genes demonstrated a reverse trend (CS16 *n*=3, CS17 *n*=4): the lowest CS16 expressing genes become highly expressed at CS17, whereas the highest expressing genes dropped their expression (Fig. 3A), which might manifest homeostatic stabilisation of molecular signalling at the end of IAHC/HSC formation. Full lists of genes within each expression pattern can be found in Table S2.

#### Hox genes dynamics

Various studies have demonstrated that HOX genes play diverse roles in haematopoietic differentiation (Bhatlekar et al., 2018). In the human, HOXA transcription factors (TFs) (*HOXA5*, *HOXA7*, *HOXA9*, *HOXA10*) were proposed to define the stemness of HSCs *in vivo* (Dou et al., 2016; Ng et al., 2016). Overexpression of HOXA TFs has been used to enhance multi-potency and self-renewal of human haematopoietic progenitors (Doulatov et al., 2013; Dou et al., 2016; Sugimura, 2019). Recently, *HOXA9* has been included in the molecular signature of the human AGM-derived HSCs (Calvanese et al., 2022). Therefore, our analysis of HOX genes is focused on the inner compartments of the Ao, VI – the site of HSC emergence – and DI. We found that all genes of the HOXA group are expressed in VI with different dynamics across the CS13-CS17 window (Fig. 3B,C; Fig. S3A,B). The expression of majority of HOXA genes peaks at CS16 in line with increased detectability of HSC at this stage (Ivanovs et al., 2011). Amongst all HOXA genes, *HOXA5*, *HOXA7*, *HOXA9*, *HOXA10* and additionally *HOXA3* showed the highest expression levels. (Fig. S3C). Stage-specific ventral enrichment of consecutive HOXA genes, *HOXA10*, *HOXA11* and *HOXA13*, was observed at CS13, CS14 and CS17 respectively (Fig. S3C).

Similar to HOXA, various HOXB genes are also broadly expressed in the AGM region (Fig. S3Ai,Aii). *HOXB2*/*3*/*5*-*9* are expressed at high relatively steady levels, whereas HOXB4 expression starts low and grows monotonically during CS13-CS17. Notably, HOXB4 which is a strong stimulator of HSC generation in the mouse (Antonchuk et al., 2002; Kyba et al., 2002; Lu et al., 2016), is one of the most ventrally enriched HOXB genes (Fig. S3Ai,C). HOXC group of genes are also expressed broadly and at variable levels, some peaking at CS16 (e.g. *HOXC6*) and some (*HOXC10*-*13*) raising monotonically through the entire CS13-CS17 period (Fig. S3Bi,Bii). Of interest, the HOXC genes showed distinctive synchronised polarisation at CS16: ventralisation of earlier (*HOXC4*-*9*) genes and dorsalisation of later (*HOXC10*-*13*) genes (Fig. S3C). The significance of dynamic complexity of HOX genes in regulating EHT/HSC development is unclear but is an intriguing issue for investigation.

#### Evolvement of the HSC molecular signature

We next sought to explore the expression dynamics of transcription factors *RUNX1*, *HOXA9*, *MLLT3*, *MECOM* and *HLF*, which were reported to be enriched in human AGM-derived HSCs (Calvanese et al., 2022). We found that expression of these TFs is high and peaks at CS16 before tapering off at CS17 (except *RUNX1*), potentially marking the end of the HSC temporal window (Fig. 3D). Of these TFs, *HLF* behaves distinctively: its initially low expression rises steeply during the CS13-CS14 transition, suggesting its early involvement in HSC formation (Fig. 3D). Notably, *HLF* is considered to be the most specific within the AGM-derived HSC signature (Calvanese et al., 2022).

The analysis of dorso-ventral polarisation revealed fluctuations in differences of expression of these genes between VI and DI within the range of ±1.25 Log2 fold change interval. Of them, only one, *HLF*, showed dramatic ventral enrichment during the CS13-CS14 transition (Fig. 3C), again pointing at its potential role in early IAHC/HSC development. Later, significant *HLF* ventral polarisation, along with *RUNX1*, was observed at CS17, whereas *HOXA9* expression tended to go down (Fig. 3C).

### Stage-specific molecular interactions in AoV during IAHC/HSC formation

**Fig. 4.**
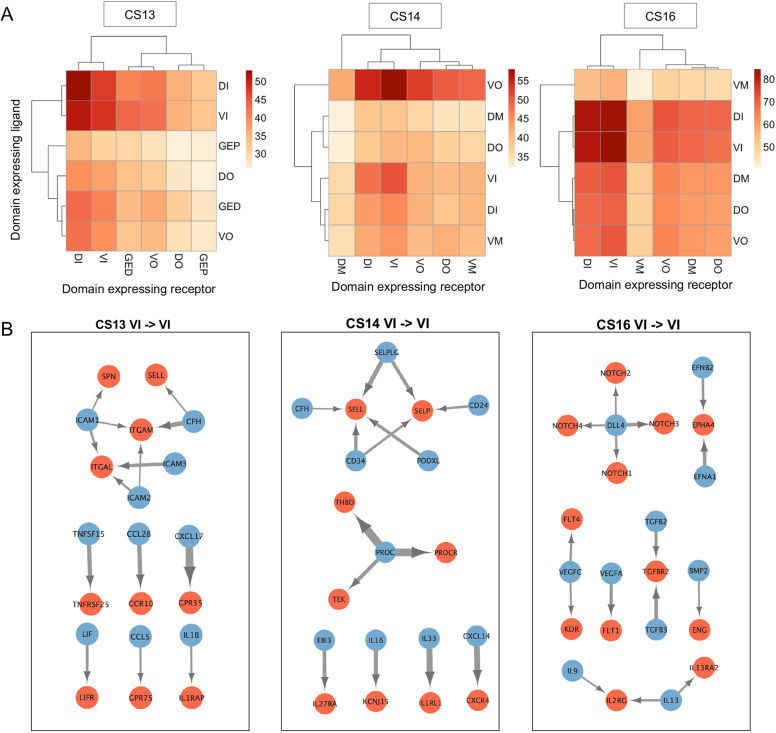
**Stage-specific molecular interactions within the AoV during IAHC/HSC formation.** (A) Heatmap of the number of predicted ligand-receptor interactions between spatial domains for CS13, CS14 and CS16 (detection threshold 0.2). DI, dorsal inner; DM, dorsal mid; DO, dorsal outer; GED, gonadal epithelium distal; GEP, gonadal epithelium proximal; VI, ventral inner; VM, ventral mid; VO, ventral outer. (B) Network map of top ligand (blue)-receptor (red) interactions for VI→VI for CS13, CS14 and CS16. Arrow width correlates with specificity score.

To better characterise potential signalling interactions between spatial domains, Network Analysis Toolkit for Multicellular Interactions (NATMI; Hou et al., 2020) was used to predict ligand-receptor interactions for LCM-seq at CS13, CS14 and for previously published LCM-seq CS16 datasets (Crosse et al., 2020). The full set of interactions between spatial domains for each dataset can be interactively explored at https://medvinsky-lab.github.io/hsc-niche-explorer/. A heatmap for predicted (potential) ligand-receptor interactions by specificity edges (see Materials and Methods) between LCM-seq spatial domains for each embryo stage is shown in Fig. 4A. For each stage, we have focused predominantly on signalling towards receptors in the VI as the site of HSC emergence.

At CS13, VI is predicted to receive the highest number of signalling ligands from itself and DI, its dorsal counterpart (Fig. 4A). Specific ligand-receptor interactions will be further labelled as follows: ligand→receptor. Network analysis of the predicted top ligand-receptor interactions shows a cluster of adhesion molecules signalling within CS13_VI including integrins (*ITGAM*, *ITGAL*) and *ICAM1*-*3* (all edge weights>0.2) (Fig. 4B). There are also several cytokine-receptor interactions within VI including CCL28→CCR10, which has a described role in promoting proliferation of primitive human HSPC (Karlsson et al., 2013). In contrast to VI ligand-VI receptor molecular interactions, the predicted DI-VI molecular interactions (Fig. S4A) do not include integrins and have fewer cytokines in line with the paucity of proinflammatory signalling described above for the CS13_DI (Fig. 1D). Although ventral signalling is pivotal for HSC development, the dorsal domain of the Ao may also contribute into this process as shown for the mouse (Souilhol et al., 2016a). For example, NODAL and INHBA, known to be involved in mesoderm patterning, fate specification and epithelial-mesenchymal transition (Green et al., 1992; Perea-Gomez et al., 2002; Shen, 2007; Yu et al., 2021), are secreted by CS13_DI. Despite having relatively few predicted interactions with CS13_VI (Fig. 4A), the GEP, which is initially adjacent to the ventral floor of the Ao, might be a crucial source of BMP4 for developing HSCs at this early stage (Fig. S4B). Distancing of the GEP from the Ao at later stages may attenuate the impact of BMP4, which may be necessary for completion of HSC maturation as shown in the mouse (Souilhol et al., 2016a).

At CS14, the VI maintains a high number of predicted interactions within itself (VI ligand→VI receptor) including again a cluster of adhesion molecules but now centred around selectins (*SELL*, *SELP*) rather than integrins (Fig. 4A,B). Such temporal changes in adhesion dynamics during CS13-CS14 might underlie cell transitions and movements during EHT, including such processes as the potential rolling of HSPCs along the Ao endothelium and trans-endothelial monocyte/macrophage migration (Perlin et al., 2017; Mariani et al., 2019). Notably, PROC (Protein C), according to NATMI, has predicted interactions with three receptors, PROCR (EPCR), THBD and TEK, within CS14_VI. PROCR is an important marker for mouse pre-HSC and human fetal liver HSCs (Zhou et al., 2016; Subramaniam et al., 2019). Given the functional importance of PROCR in HSCs (Gur-Cohen et al., 2016; Chagraoui et al., 2019), the spike of PROC signalling within CS14_VI might contribute to the onset of IAHCs and development of definitive HSCs. Another contrast to CS13 is that at CS14 the highest number of signalling ligands being received by the VI are predicted to derive from the VO, suggesting its impact on initiation of IAHC/HSC specification (Fig. 4A). However, as VO is separated from the VI by the VM layer, impactful VO-derived molecules are likely to be low molecular weight diffusible proteins (usually below 45 kDa) capable of migrating significant distances. Among these CS14_VO-derived ligands is LIF, which can potentially interact with LIFR expressed in CS14_VI (Fig. S4C). LIF is a pleiotropic cytokine that is a component of ‘IL2 STAT5 signalling’, ‘TNFA signalling via NfκB’ and ‘KRAS’ hallmark pathways, which are also enriched in the CS14 VI. Another CS14_VO-derived ligand is SPP1 (osteopontin), predicted to interact with CD44, an EHT marker (Oatley et al., 2020) enriched within the CS14_VI. CD44 is also integrated in ‘IL2 STAT5 signalling’, ‘KRAS’ and ‘Epithelial mesenchymal transition’ pathways (Fig. S4C; Fig. 2B). Altogether, our data suggest that in addition to proximal interactions, more distal sub-aortic mesenchyme contributes towards HSC development at CS14.

At CS16 the ligand-receptor predicted interactions are again most abundant within VI (*n*=3) (Fig. 4A), although these are distinct from VI→VI signalling at CS13 and CS14 (Fig. 4B). There is a further transition in the adhesion profile within CS16_VI: ephrins become top adhesion molecules instead of previously dominant integrins and selectins. Classical signalling pathways involved in angiogenesis and HSC generation are top predicted interactions, including Notch signalling (DLL4→NOTCH1-4), TGF beta/BMP signalling (TGFB2/3→TRFBR2 and BMP2→ENG) and VEGF signalling (VEGFA→FLT1 and VEGFC→FLT4/KDR). Overall, our analysis shows that signalling networks during IAHC/definitive HSC formation in the AoV undergo gradual temporal changes, potentially underpinning the multi-step process of HSC development**.**

### Characterisation of haematoendothelial and stromal populations

**Fig. 5.**
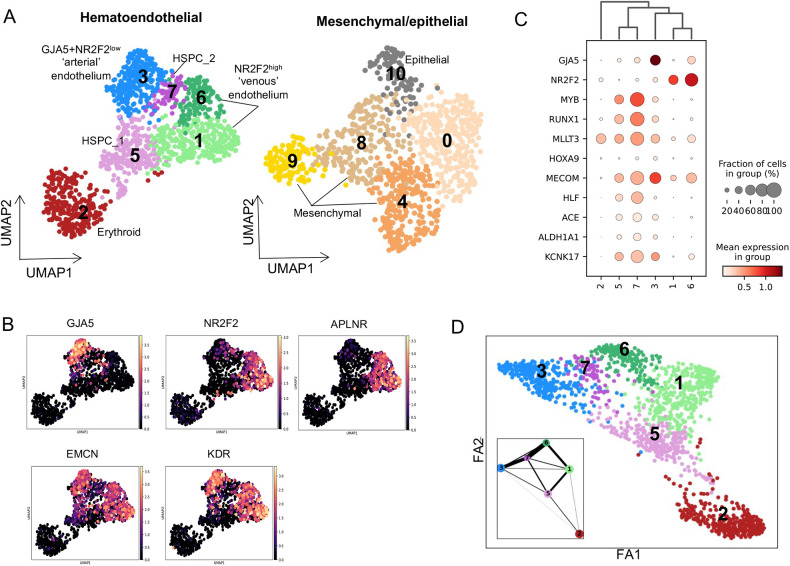
**Characterisation of haematoendothelial and stromal populations of the Ao.** (A) UMAPs for haematoendothelial and mesenchymal/epithelial populations from the dorsal aortae of CS13-CS15 embryos. (B) Expression of endothelial markers across the haematoendothelial populations. (C) Dot plot of mean expression of endothelial (GJA5, NR2F2), developing HSC (MYB, RUNX1, MLLT3, HOXA9, MECOM, HLF, ACE) and haematogenic endothelium (ALDH1A1, KCNK17) markers for each cluster in A. (D) Force directed graph and PAGA plot (insert) predicting lineage relationships between clusters from UMAP in A.

In order to resolve signalling from the spatial transcriptome datasets to discrete cell populations, single cell transcriptomics data obtained from a CS13 Ao was integrated with previously published 10x Genomics single-cell datasets from selected CS13-CS15 populations (Zeng et al., 2019) (Fig. 5A; Fig. S5A,B). The resultant dataset gives a broad overview of the Ao niche with enrichment of endothelial and haematopoietic progenitor populations and consists of the following five samples: ‘CS13_Aorta’ (total live 7AAD^−^ cells); ‘CS13_Aorta_b’ (total live 7AAD^−^CD235a^−^ erythroid depleted cells), ‘CS13_Aorta_Endo’ and ‘CS14_Aorta_Endo’ (live 7AAD^−^CD235a^−^CD45^−^CD34^+^ endothelial cells), respectively and ‘CS15_Aorta_HSPC’ (7AAD^−^CD235a^−^CD45^+^CD34^+^ HSPCs).

These data were organised in two separate datasets visualised by UMAP: (1) endothelial/haematopoietic and (2) mesenchymal/epithelial populations of the Ao proximal stromal layers (Fig. 5A). Clustering analysis followed by marker identification determined population identities. Within the endothelial/haematopoietic dataset the endothelial CDH5-positive cells were split into two major types expressing classical arterial markers *GJA5* and *GJA4* (cluster 3; Cl.3) and venous markers *NR2F2* and *APLNR* (Cl.1 and Cl.6) (Fig. 5B). However, this arterial-venous split is not clearly demarcated due to promiscuous expression of these and other markers (Fig. 5C), similar to our previous observation for CS16 (Crosse et al., 2020). For convenience, Cl.3 will be termed ‘arterial’ and Cl.1 and Cl.6 as ‘venous’, based on predominant expression of corresponding markers.

HSPCs are represented by Cl.5 and Cl.7, both expressing developing HSC markers *MYB*, *RUNX1*, *MLLT3*, *MECOM*, *HLF* and *ACE*, with higher expression consistently in Cl.7 (Fig. 5C) (Julien et al., 2016; Calvanese et al., 2022). Compared with HSPC Cl.5, HSPC Cl.7 demonstrates a considerable abundance of molecular pathways, including several proinflammatory pathways (‘TNFA signalling via NfκB’, ‘Estrogen response late’ and ‘Interferon gamma response’; Fig. S5C). HSPC Cl.7 also shows upregulation of signalling associated with cell proliferation (‘Mitotic spindle’, ‘G2M checkpoint’, ‘E2F targets’ pathways). Accordingly, Cl.7 cells are predominantly cycling whereas Cl.5 cells are mainly G0/G1 (Fig. S5D). The third haematopoietic Cl.2 (*BLVRB*, *GYPC*) is erythroid. The remaining non-haematoendothelial (‘niche’) populations include Cl.4 (*TAGLN*, *TGFB1I1*, *INHBA*) smooth muscle cells, Cl.10 (*APOA1*, *EPCAM*, *MPC2*) epithelial cells and three undetermined cell populations: Cl.8 (*SOST*, *CARD10*, *FRMD3*), Cl.9 (*SMC4*, *GTSE1*, *TOP2A*) and Cl.0 (Fig. 5A, Fig. S5E). Cl.0 is a more ambiguous stromal sub-population with no strong expression of marker genes which may represent an earlier less-differentiated cell population.

The different samples contribute differentially across the sub-populations as expected due to different initial sorting strategies (Fig. S5B). As the only sample without erythroid depletion, CS13_Aorta is the predominant contributor to erythroid Cl.2. Along with CS13_Aorta_b (7AAD^−^CD235a^−^) it also contributes to the majority of mesenchymal/epithelial cells., Meanwhile, CS13_Aorta_Endo and CS14_Aorta Endo (7AAD^−^CD235a^−^CD45^−^CD34^+^) contribute predominantly to endothelial clusters ‘arterial’ Cl.3 and ‘venous’ Cl.6. Interestingly, these samples have very little contribution to endothelial Cl.1 (which predominantly comprises CS13_Aorta and CS13_Aorta_b cells) indicating that this is a low CD34-expressing endothelial population. Finally, as expected, CS15_HSPC (7AAD^−^CD235a^−^CD45^+^CD34^+^) contributes predominantly to HSPC Cl.5 and Cl.7 with some additional contribution to ‘arterial’ Cl.3.

Using partition-based graph abstraction (PAGA) and force directed maps (Wolf et al., 2019) (Fig. 5D), we inferred predicted lineage relationships between clusters. HSPC Cl.5 and Cl.7 in this dataset showed lineage relationships with both *GJA5^+^NR2F2*^low^ arterial Cl.3 and *APLNR^+^NR2F2*^high^ venous Cl.1 and Cl.6 (Fig. 5D). However, whereas HSPC Cl.7 has strong predicted lineage relationships to both arterial Cl.3 and venous Cl.6, HSPC Cl.5 is more strongly linked with venous-like Cl.1 and significantly weaker with arterial Cl.3 (Fig. 5D). That said, a positive gradient of expression of developing HSC markers (*MYB*, *RUNX1*, *MLLT3*, *MECOM*) and haematogenic endothelium makers (*ALDH1A1*, *KCNK17*) is clearly observed between arterial Cl.3 and Cl.5/Cl.7 (Fig. 5C). The exact relationship between arterial/venous and HSC/HSPC emergence *in vivo* requires further investigation.

### Signalling networks across the HSC developmental niche

**Fig. 6.**
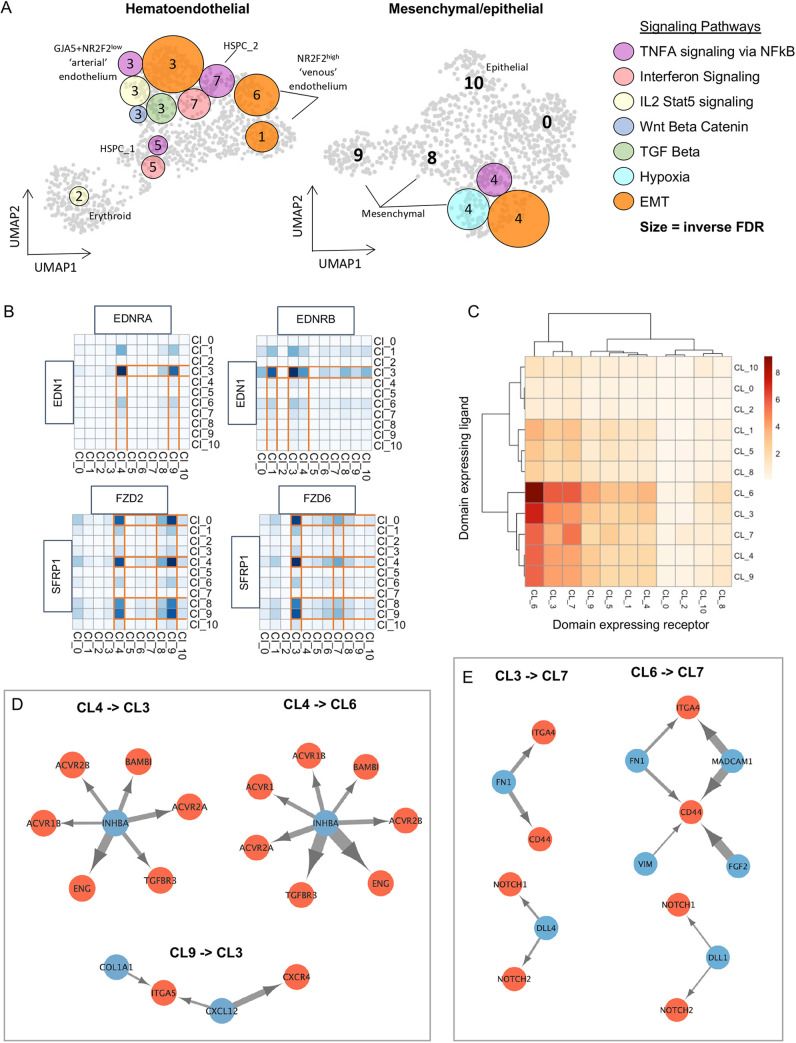
**Resolved signalling networks across the Ao developmental niche.** (A) Mapping of selected ventrally enriched signalling pathways to clusters on CS13-CS15 dorsal aortae UMAPs from Fig. 5A. Circle colour matches the pathway shown in the key. Circle size correlates with significance (size increases inverse to FDR). (B) Heatmap of predicted ligand-receptor signalling by expression and specificity edges for the indicated genes across all populations of the CS13-CS15 dorsal aorta UMAP. (C) Heatmap of the number of predicted ligand-receptor interactions between UMAP clusters (detection threshold 0.2). (D,E) Network map of selected top ligand (blue)-receptor (red) interactions between indicated clusters.

Sections ‘CS13 to CS14 as a key checkpoint in HSC generation’ and ‘Pathway dynamics in AoV during CS13-CS17’ highlighted signalling pathways potentially involved in HSC generation owing to their ventralised enrichment. Using GSEA of the single cell datasets, these pathways were resolved to discrete populations (Fig. 6A). Some of them, such as ‘IL2 STAT5 signalling’, ‘Wnt beta catenin’, ‘TGF beta’ (all Cl.3) and ‘Hypoxia’ (Cl.4) were resolved to unique clusters. Others were enriched across several cell types such as ‘TNFA signalling via NfκB’ (stromal Cl.4, HSPC Cl.5 and Cl.7, and arterial Cl.3) and ‘Epithelial mesenchymal transition’ (stromal Cl.4, arterial Cl.3, and venous Cl.1 and Cl.6). Using NATMI, we resolved ligand-receptor pairs of interest within the above pathways which may mediate interactions between cell clusters (Fig. 6B). For example, EDN1 supporting HSC emergence in the human embryo (Crosse et al., 2020) is part of the ventrally enriched ‘TNFA signalling via NfκB’ pathway in the CS14 AoV (Fig. 1C). Analysis of predicted relationships in CS14 AoV showed signalling of *EDN1* predominantly from arterial Cl.3 towards mesenchymal Cl.4 and Cl.9 via receptor *EDNRA* and back to itself as well as towards the endothelial Cl.1 via receptor *EDNRB* (Fig. 6B), recapturing our previous finding for CS16 (Crosse et al., 2020). Similarly, the Wnt antagonist *SFRP1* downregulated in the CS14 AoV compared with CS13 AoV (Fig. 2A) was predicted to have two directions of signalling through alternate receptors: predominantly stromal-derived (Cl.0, Cl.4, Cl.8 and Cl.9) *SFRP1* is predicted to signal towards arterial Cl.3 and HSPC Cl.7 through *FZD6*, and signal back to the same stromal compartments in an autocrine manner through *FZD2* (Fig. 6B).

NATMI was also used to predict ligand-receptor interactions between populations in an unbiased way. Venous Cl.6 (followed by arterial Cl.3 and HSPC Cl.7) consistently had the highest number of receptors with predicted ligand pairs from all populations across the dataset (Fig. 6C). This is indicative of skewed directional signalling within the AGM niche towards vascular remodelling and EHT. A closer look at signalling molecules directed towards the endothelium identified *INHBA* as a potential key player mediating signalling between mesenchymal Cl.4 and both venous Cl.6 (*ENG*, *ACVR2A*, *ACVR2B*, *ACVR1*, *ACVR1B*, *TGFBR3*, *BAMBI*) and arterial Cl.3 (*ENG*, *ACVR2A*, *ACVR2B*, *TGFBR3*, *BAMBI)* (Fig. 6D). A member of ‘TNFA signalling via NfκB’, ‘Epithelial mesenchymal transition’ and inflammatory response pathways, which are upregulated in the CS14 AoV compared with CS13 (Fig. 2B), INHBA may be an important signalling player during the CS13-CS14 transition. We also found that mesenchymal Cl.9 secretes CXCL12, predictively targeting the arterial Cl.3 via the CXCR4 receptor (Fig. 6D). This is of interest as CXCL12 is involved in maintenance and function of HSCs *in vivo* (Lapidot and Kollet, 2002; Wright et al., 2002; Sugiyama et al., 2006).

Ligand-receptor pairs were also predicted between endothelial Cl.3 and Cl.6 and HSPC Cl.7 (Fig. 6C,E), which may reflect involvement of autocrine signalling during EHT and support previous reports on the role of endothelium in the AGM niche (Butler et al., 2010; Heck et al., 2020; Hadland et al., 2022). Interactions of endothelial Cl.3 and Cl.6 with HSPC Cl.5 are also predicted but less prominent. CD44, which is upregulated in CS14_VI (Fig. 1C) and marks EHT (Oatley et al., 2020), is enriched in HSPC Cl.7 and can potentially mediate signalling from arterial Cl.3 via secreted FN1 ligand, and from venous Cl.6 via FN1, MADCAM1, VIM and FGF2 ligands.

Our analysis revealed an interesting feature of Notch signalling in the AoV. Notch signalling is essential for HSC development (Kumano et al., 2003; Hadland et al., 2004; Gama-Norton et al., 2015; Uenishi et al., 2018) but needs to be downregulated by the end of HSC maturation in the AGM region, which may be mediated, at least partly, by Notch ligands with different strength (Bigas and Espinosa, 2012; Souilhol et al., 2016b). We found that Notch ligands *DLL4* and *DLL1* are expressed separately by arterial and venous endothelia (Cl.3 and Cl.6, respectively) (Fig. 6E), raising the possibility that HSC maturation is driven by differential spatially and temporally defined contacts with these endothelial compartments. NATMI-predicted signalling interactions between all populations in this dataset can be interactively explored at https://medvinsky-lab.github.io/hsc-niche-explorer/.

## DISCUSSION

Embryonic HSC precursors emerge within the complex signalling milieu of the AGM region in the Ao (Medvinsky and Dzierzak, 1996; de Bruijn et al., 2002). Significant progress has been made in identifying key genetic regulators of HSC development in model organisms (Ciau-Uitz et al., 2016; Weijts et al., 2021). Owing to ethical reasons and scarcity of samples, HSC development in humans has been much less studied than in model organisms. Progress in analysis of mechanisms underlying human HSC development is further hampered due to differences with model organisms as well as failure to establish human AGM cultures capable of recapitulating HSC development (Easterbrook et al., 2019). As a result, attempts to derive bona fide HSCs from pluripotent stem cells without genetic reprogramming have been unsuccessful (Sugimura et al., 2017; Fidanza and Forrester, 2021). Analysis of the data-rich resource generated in this study revealed a plethora of molecular processes with a potential role in HSC development that may serve to fill the gaps in our understanding.

The evidence that IAHC and HSC development in the Ao is ventralised sparked interest in spatially polarised expression of genes in the AGM region (Tavian et al., 1996; Marshall et al., 2000; Taoudi and Medvinsky, 2007; Peeters et al., 2009; Ivanovs et al., 2014). The spatial asymmetry of IAHC/HSC development may result from differential signals emitted from the surrounding stroma (Souilhol et al., 2016a; McGarvey et al., 2017; Crosse et al., 2020) and also from differences in origins of the dorsal and ventral portions of the Ao (Pardanaud et al., 1996). Combined spatial and single cell transcriptomics proved to be a powerful approach for analysing HSC development. It led to identification of factors supporting HSC development, such as the ADM-RAMP2 ligand-receptor pair between the niche and emerging HSPC (Yvernogeau et al., 2020), endothelin 1 secreted molecule (Crosse et al., 2020) and of the molecular signature enriched in developing human HSC (Calvanese et al., 2022).

Here, based on the understanding that HSC development in the mouse model is step-wise over a temporal window (Taoudi et al., 2008; Rybtsov et al., 2011, 2014; Zhou et al., 2016; Hadland et al., 2022), we have captured a dynamic representation of polarised signalling throughout the human CS13-CS17 HSC developmental period. We had a particular interest in the CS13-CS14 transition, when IAHC and definitive HSCs first emerge. Significant dorso-ventral polarisation was detectable already at CS13, prior to emergence of definitive IAHC/HSCs, which is reflected in upregulation of respective markers (e.g. *MYB*, *PROCR*, *HLF*, *CD44* and *MECOM*; *HLF* being important in the molecular signature of developing HSCs) (Calvanese et al., 2022). Simultaneously, we observed a striking increase in signalling complexity in AoV, likely reflecting both vascular remodelling and EHT. One of the upregulated signalling pathways was EMT, which may contribute to EHT via an integral role of molecules such as CD44 (Xu et al., 2015; Oatley et al., 2020). TGF beta signalling, which underlies both EMT and EHT, is also upregulated at CS14 (Lamouille et al., 2014; Monteiro et al., 2016; Lempereur et al., 2018; Thambyrajah et al., 2018). In addition, the spike of signalling complexity at CS14 also included BMP signalling and hypoxia, known to be involved in HSC development in model organisms (Wilkinson et al., 2009; Imanirad and Dzierzak, 2013; McGarvey et al., 2017; Wang et al., 2022).

Proinflammatory signalling was also upregulated at CS14, intensifying further through the HSC developmental window, which is consistent with observations in zebrafish and mouse models (Espín-Palazón et al., 2014; Espín-Palazón and Traver, 2016; Collins et al., 2021). In human AoV, the proinflammatory pathways included ‘IL2 STAT5 signalling’, ‘IL6/JAK/STAT3 signalling’, ‘TNFA signalling via NfκB’, ‘Interferon alpha response’ and ‘Interferon gamma response’. Although primitive myeloid cells secrete proinflammatory molecules in the AGM region (Li et al., 2014; Mariani et al., 2019), we found that ‘TNFA signalling via NfκB’ also spanned several non-haematopoietic populations including arterial endothelium and mesenchymal stromal cells, while ‘IL2 STAT5 signalling’ was also resolved to the arterial endothelium. Thus, haematogenic endothelium and developing HSCs potentially receive proinflammatory signals from multiple AGM cell populations.

Overall, significant increase in complexity of signalling suggests that the CS13-CS14 transition is a key check-point potentially underlying the appearance of IAHCs and definitive HSCs. Dynamic changes within the VI compartment during the HSC developmental window included expression waves of integrins, selectins and ephrins observed sequentially at CS13, CS14 and CS16 stages. We do not have a clear picture of migration pathways of mammalian HSCs and adhesion mechanisms underlying their mobility, although β1 integrins were shown to be essential for colonisation of the embryonic liver (Hirsch et al., 1996). Selectins act as substrates for HSPC rolling and migration in the human and zebrafish (Greenberg et al., 2000; Esain et al., 2015) and ephrins mediate haematopoietic cells interactions with adult bone marrow stroma (Ting et al., 2010; Kwak et al., 2016). How dynamic changes in adhesion impact IAHC formation/HSC maturation and migration behaviour requires further elucidation.

We also looked into dynamic expression of HOX genes during HSC emergence, as these genes play different important roles during haematopoietic development and differentiation (Bhatlekar et al., 2018). We found that all genes of HOXA, B and C groups are expressed in the VI during CS13-CS17 at different levels and with different dynamics. What the role of this complex spatiotemporal pattern of HOX gene expression is in HSC development remains an open question. Our resource enables various hypotheses to be raised and tested.

Endothelial cells give rise to HSCs and progenitor cells and concurrently are an important component of the HSC niche, both in the embryo and in the adult (Kenswil et al., 2021; Hadland et al., 2022; Yu et al., 2022). Our combined spatial and single cell transcriptomics analysis revealed heterogeneity in the composition of the Ao endothelium with some overlap between arterial and venous molecular characteristics, in line with our previous report for CS16 (Crosse et al., 2020). HSPCs in the Ao were represented by two distinct populations, which differed strikingly by proliferative state and proinflammatory signature. Our analysis supports a broadly accepted view on lineage relationship of HSPCs with the arterial endothelium (Clements and Traver, 2013; Park et al., 2018). However, we also observed a strong predicted relationship with the venous-like endothelium, as we have previously described at CS16. Notably, the venous population showed the most abundant ligand-receptor interactions (in both directions) with itself, arterial-like cells, HSPCs and stromal cells, suggesting an integral role of the venous compartment within the AGM niche. The precise relationship and interactions between arterial/venous endothelium within the human dorsal aorta and HSC/HSPC requires further detailed investigation.

In summary, we have generated a data-rich resource, which deconvolutes the signalling landscape of the developing human HSC niche at the spatial and cell population levels over the period of HSC generation in the human AGM region. This provides a hypothesis-generating platform for functional investigation into cell lineage relationships and mechanisms of HSPC generation. An interactive application presented here enables efficient exploration of this resource. Researchers can cross-reference with their preferred model organisms’ datasets to facilitate identification of evolutionarily conserved and disparate mechanisms of HSC development. This resource may provide useful insights into *ex vivo* derivation of transplantable HSC for clinical needs.

## MATERIALS AND METHODS

### Human embryonic material

Human embryonic samples of CS13-CS14 were provided by the MRC Centre for Reproductive Health and by the Joint MRC/Wellcome (MR/R006237/1) Human Developmental Biology Resource (https://www.hdbr.org/). This study was approved by the Lothian Research Ethics Committee. The embryos were obtained immediately after elective termination of pregnancy for which each patient gave informed consent in writing. Embryos were either used immediately as fresh tissue or flash frozen in Optimal Cutting Temperature (OCT) compound and stored at −80°C.

### Laser capture microdissection

Human embryos embedded in OCT stored at −80°C were equilibrated to −24°C and sectioned in a caudal-to-rostral direction using a cryotome FSE cryostat (Thermo Fisher Scientific). Frequent checks under the microscope verified the level reached along the rostral-caudal axis as defined by anatomical landmarks. For both CS13 and CS14, transverse 10 µm sections were taken between the most caudal appearance of the midgut loop and the caudal appearance of the liver (Fig. S1A). Once the appropriate level had been reached, cryosections were transferred onto nuclease-free polyethylene napthalate (PEN) membrane slides (Zeiss). At intervals, a sister section would be transferred to a SuperFrost slide for future validation of ventral IAHCs by immunohistochemical analysis. For visualisation during LCM, sections were stained using a rapid Haematoxylin and Eosin staining protocol: 3 min 70% ethanol, 1 min H_2_O, 4 min Mayer's Haematoxylin Solution (Sigma-Aldrich), 2 min tap H_2_O, 15 s Eosin Y (Sigma-Aldrich), 1 min 70% ethanol, 1 min 90% ethanol and 3 min 100% ethanol. All H_2_O was treated with diethyl pyrocarbonate (DEPC) and all reagents were pre-cooled in ice (except 100% ethanol, which was room temperature).

The laser capture microscope used was the PALM microbeam (Zeiss). The microscope and surrounding area were sprayed down with RNaseAWAY^®^ (Sigma-Aldrich). Sections were viewed and microdissected in brightfield using a 10× objective. The microdissected regions were collected into the caps of AdhesiveCap 500 opaque (Zeiss) 500 μl PCR tubes. Then, 15 μl lysis buffer (Nichterwitz et al., 2016) [0.2% Triton X-100 (Sigma-Aldrich), 2 U/μl RNase inhibitor (Takara), phosphate buffer solution (PBS)] was added directly on top of the dissected tissue in the tube caps and the tubes were closed in an inverted position.

### Immunofluorescence and confocal imaging

Embryo sections, obtained as described above, were fixed in cold 4% paraformaldehyde (Sigma-Aldrich) for 10 min and stained as follows: 3× wash in PBS, 5 min each; 10 min permeabilisation in PBS/0.5% Triton X-100 (Sigma-Aldrich); 2×5 min PBS wash; 30 min PBS/10% fetal calf serum (FCS) protein block; overnight incubation with a primary antibody diluted in PBS/2% FCS; 2×5 min PBS wash; 2 h incubation with secondary antibody diluted in PBS/2% FCS at room temperature; 2×5 min PBS wash; 5 min 30 nM DAPI (Thermo Fisher Scientific) incubation; 1×5 min PBS wash; mount in Prolong Gold Antifade (Thermo Fisher Scientific) and coverslip of thickness No. 1.5 (VWR). Sections were stained with mouse monoclonal anti-CD144 (1:50, BD Biosciences, Cat# 555661), rabbit monoclonal anti-RUNX1 (1:100, Abcam, Cat# ab92336) primary antibodies and goat polyclonal anti-mouse IgG (H+L) AF488 (1:200, Thermo Fisher Scientific, Cat# A-11001) and donkey polyclonal anti-rabbit IgG (H+L) AF647 (1:200, Abcam, Cat# ab150075) secondary antibodies. Negative controls (no primary antibodies) were also included.

### RNA-seq library preparation

The previously published LCM-seq protocol (Nichterwitz et al., 2016) was used for RNA-seq library preparation from LCM material in lysis buffer. LCM caps were vortexed for 15 s and spun in a tabletop centrifuge (8000 ***g***) for 5 min. Then, 5 μl lysate was added to 2 μl 10 mM dNTP mix (Thermo Fisher Scientific) and 1 μl 10 μM oligodT (5′-AAGCAGTGGTATCAACGCAGAGTACTTTTTTTTTTTTTTTTTTTTTTTTTTTTTTVN-3′; Integrated DNA Technologies). This was vortexed briefly and spun in a microcentrifuge for 30 s (700 ***g***) then incubated at 72°C for 3 min and immediately snap cooled on ice. To each reaction, 2 μl SSRTIV 5× buffer, 0.5 μl 100 mM DTT, 0.5 μl 200 Uμl^−1^ SSRTIV (all Thermo Fisher Scientific), 2 μl 5 M betaine (Sigma-Aldrich), 0.1 μl 1 M MgCl_2_ (Sigma-Aldrich), 0.25 μl 40 Uμl^−1^ RNase inhibitor (Takara) and 0.1 μl 100 μM TSO-LNA-oligo 5′-AAGCAGTGGTATCAACGCAGAGTACATrGrG+G-3′; Exiqon) was added. The reverse transcription reaction was performed in a thermal heat cycler with the following conditions: 90 min 42°C, ten cycles of 2 min at 50°C, 2 min at 42°C, and then 15 s at 70°C. For the amplification reaction, 12.5 μl 2× KAPA HiFI Hotstart Mix (KAPA Biosystems), 0.2 μl 10 μM ISPCR primers (5′-AAGCAGTGGTATCAACGCAGAGT-3′; Integrated DNA Technologies) and 2.3 μl nuclease-free H_2_O (Invitrogen) was added to each reaction. This was heat cycled as follows: 3 min at 98°C, 18 cycles of 20 s at 98°C, 15 s at 67°C, 6 min at 72°C, and then 5 min at 72°C. After bead purification using AMPure XP beads (Beckman Coulter), the concentration of the cDNA library was measured with an Agilent 2200 TapeStation using the High Sensitivity DNA 5000 kit (Agilent). Then, 1 ng of cDNA from this reaction was amplified and barcoded using the Nextera XT DNA sample preparation kit and Nextera XT index kit (Illumina) following the manufacturer's protocol. The libraries were purified again using AMPure XP beads, analysed on the 2200 TapeStation using the High Sensitivity DNA 100 Kit and quantified using the Qubit fluorometer and Qubit dsDNA HS Assay Kit (Thermo Fisher Scientific).

For 10x single cell sequencing, the dorsal aorta was dissected from a CS13 human embryo and dissociated into single cells in 1 mg/ml Collagenase-Dispase (Roche) and 0.12 mg/ml of DNase I (Roche) for 35 min in a 37°C rotating water bath. 10x libraries were prepared according to the manufacturer's guidelines (Single Cell 3′ Reagent Kit v2, 10x Genomics).

### RNA-seq

All RNA-seq was carried out at Edinburgh Genomics on a NovaSeq SP Flow Cell (Illumina) generating 50 base pair (bp) or 75 bp paired end reads. LCM-seq samples were sequenced at a read depth of ∼29 million reads per sample. The 10x sample was sequenced on a NovaSeq S1 Flow Cell (Illumina) with a 26/8/91 cycle set up at a read depth of ∼170,000 reads per cell.

### LCM-seq transcriptome analysis

Sequencing read quality was evaluated using FastQC, which assessed Phred quality score, adaptor contamination, GC content and duplicate levels. Illumina adaptor sequences were removed using Flexbar (Dodt et al., 2012). The alignment of reads to the human reference genome hg38 (Ensembl version 85) was performed using STAR (Dobin et al., 2013) and SAMtools (Li et al., 2009) was employed to sort and index the aligned reads. The read fragments per gene were counted and a matrix of reads per gene was generated using the BEDtools multicov function.

In R, the tool DESeq2 (Love et al., 2014) was used for differential gene expression analysis to determine fold changes in expression between different spatial domains while controlling for per embryo batch effects. The *P*-value was a Wald test statistic for differential expression between two domains. The likelihood ratio test was used to model gene dynamics across CS13-CS17 ventral domains. DEGreport was used to cluster genes differentially expressed along the CS13-CS17 temporal axis into groupings based on patterns of expression (https://www.bioconductor.org/packages/devel/bioc/html/DEGreport.html). *P*-values were adjusted for by multiple hypothesis correction with the Benjamini and Hochberg method to produce adjusted *P*-values (*P*.adj). Genes were considered significant with a *P*.adj<0.05. R packages EnhancedVolcano, pheatmap and ggplot2 were used to make plots visualising gene expression. Genes were determined to encode secreted factors, receptors or transcription factors with an unbiased approach using Uniprot: KW-0964, GO: 0038023 and GO: 0003700 terms respectively. Some overlap between Uniprot: KW-0964 and GO: 0038023 is explained by the fact that transmembrane cell surface receptors are secreted.

GSEA was carried out using the tool from the Broad Institute (Subramanian et al., 2005) using the Hallmark gene sets to identify pathways. Genes were input as a ranked list by Wald statistic and pathways were considered significant with a false discovery rate (FDR) of <0.1.

### Single cell analysis

CS13 dorsal aorta single cell data was processed using the Cell Ranger 2.1.0 (10x Genomics) analysis pipeline by aligning reads to GRCh38 human transcriptome (Ensembl). The ScanPy pipeline (Wolf et al., 2018) was used to explore and integrate the dataset with other dorsal aorta single cell datasets spanning CS13-CS15 from a publicly available resource (Zeng et al., 2019). For all datasets, cells with less than 200 genes and genes that were in less than three cells were filtered out. Cells with a percentage of mitochondrial genes >1 were also filtered out. Contaminating HB genes were also removed from the CS13 dorsal aorta dataset. The reads per cell were normalised and logarithmised. The variance effects of total counts per cell, cell cycle and mitochondrial genes were regressed out. Each dataset was subset to include only genes with highly variable expression. The tool Scanorama (Hie et al., 2019) was used to integrate datasets. Then, nearest neighbours was computed using ten principal components and the neighbourhood graph was embedded in two dimensions in a Uniform Manifold Approximation and Projection (UMAP) (Becht et al., 2018). Clustering of sub-populations within the UMAP was made using the Leiden algorithm (Traag et al., 2019). Following cluster identification by gene signature and any known identities from prior sorting strategies, the data were then subset into two parts to include haematopoietic and endothelial populations in one subset and stromal and epithelial populations in the second. PAGA (Wolf et al., 2019) was used to make lineage inferences from the neighbourhood graphs.

### Predicted ligand-receptor signalling analysis

The tool NATMI (Hou et al., 2020) was used for predicting ligand-receptor interactions across spatial domains and single cell clusters, and is the foundation for the web interactive exploration tool. The internal default ligand-receptor database ‘lrc2p’ was used, which has literature-supported ligand-receptor pairs – please see supplementary data 1 from the original NATMI publication (Hou et al., 2020) for literature references for each predicted ligand-receptor pair. Weight values of type ‘mean’ and a detection threshold of 0.2 were used. Interactions based on specificity (the mean expression of the ligand/receptor in a spatial domain or cluster divided by the sum of the mean expression of that ligand/receptor across all domains/clusters) were considered rather than interactions based on expression level as this gives a better resolution of differential contributions of the different populations.

## Supplementary Material

10.1242/develop.201972_sup1 Supplementary information

Table S1. Full lists of differentially expressed genes for comparisons described in the manuscripts (CS13 Ventral vs Dorsal, CS14 Ventral vs Dorsal, CS13 vs CS14) and differentially enriched pathways for the same comparisons (GSEA = gene set enrichment analysis). In each comparison title, the first variable corresponds to positive values. BaseMean = mean normalised expression across both conditions.

Table S2. Full list of significant genes (p.adj < 0.05) within each grouped dynamic pattern of expression across CS13 – CS17, corresponding to section ‘Pathway dynamics in AoV during CS13-CS17’.
