# Supplementary material for: An interactive resource of molecular signalling in the developing human haematopoietic stem cell niche
Source: Development. 2023 Dec 6;150(23):dev201972. doi: 10.1242/dev.201972 (PMC10730088; doi:10.1242/dev.201972)
Supplement: Supplementary information [file develop-150-201972-s1.pdf]

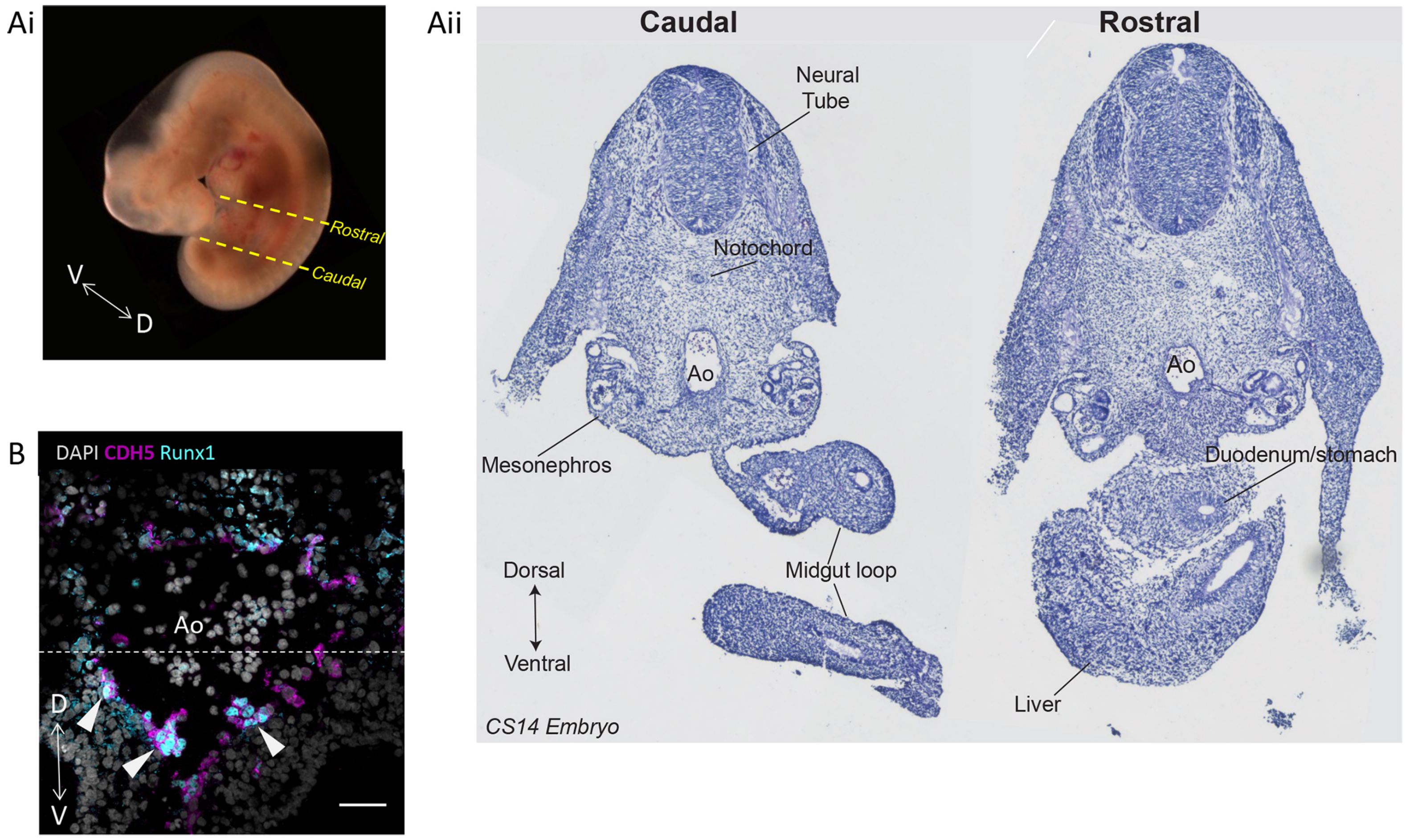

Fig. S1. (related to figure 1): Ai: A CS14 embryo with the regions of the mid trunk sectioned for laser capture microdissection indicated between the two dotted lines. Aii: Representative images of anatomical landmarks used for identification of the region to take for laser capture microdissection from a CS14 embryo; the caudal appearance of the midgut loop = most caudal, the caudal appearance of the liver and duodenum = most rostral. B: Transverse section of the dorsal aorta from a CS14 embryo showing CDH5+Runx1+ intra-aortic haematopoietic clusters exclusively in the ventral portion (below the dotted line). Scale bar is 50 μm. D = dorsal, V = ventral.

Development • Supplementary information

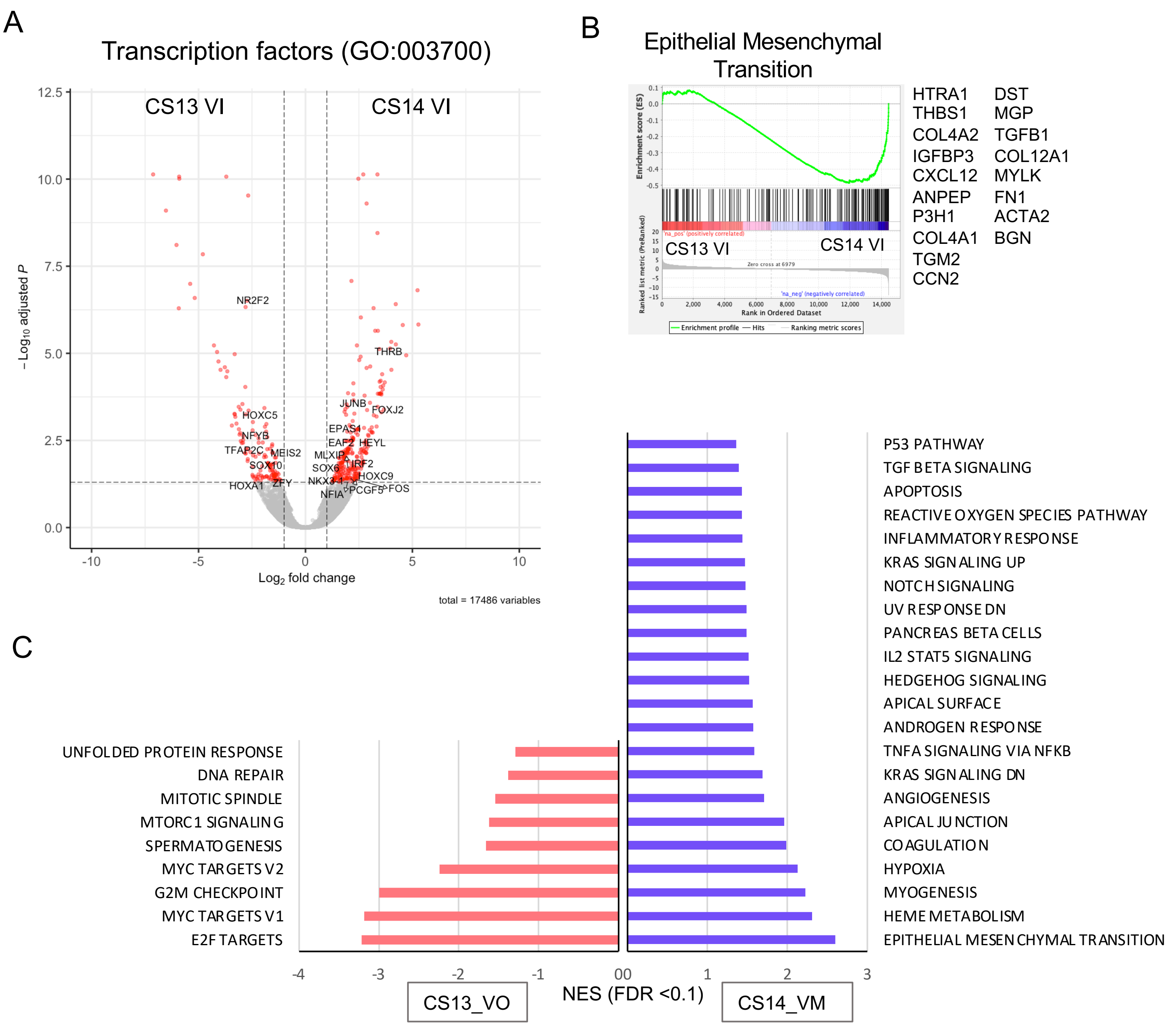

Fig. S2. (related to figure 2): A: Differentially expressed genes between the CS13 VI (n=2) and CS14 VI (n=2) with transcription factors highlighted (p.adj<0.05). B: Enrichment plot for CS13 VI vs CS14 VI 'Epithelial Mesenchymal Transition' pathway and contributing genes (significantly differentially expressed, p.adj<0.05). C: Normalized enrichment score for pathways from differentially expressed genes for CS13 VO vs CS14 VM (FDR < 0.1).

Development • Supplementary information

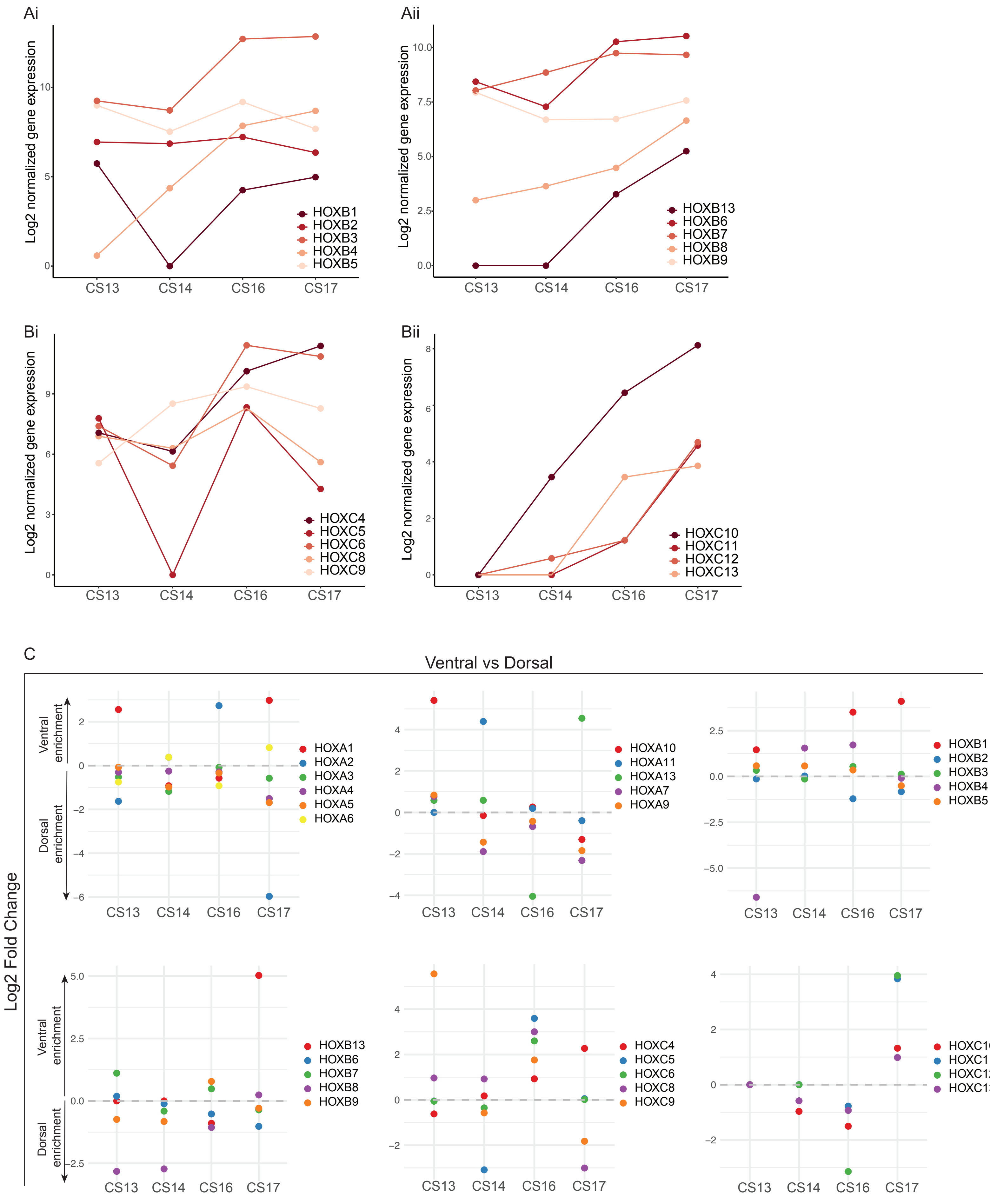

Fig. S3. Related to figure 3. A-B: Dynamics of normalised gene expression of (A) HOXB and (B) HOXC genes across CS13 – CS17 ventral vessel wall. C: Log2 fold change of HOX and developing HSC genes mean expression in ventral vs dorsal vessel wall.

Development • Supplementary information

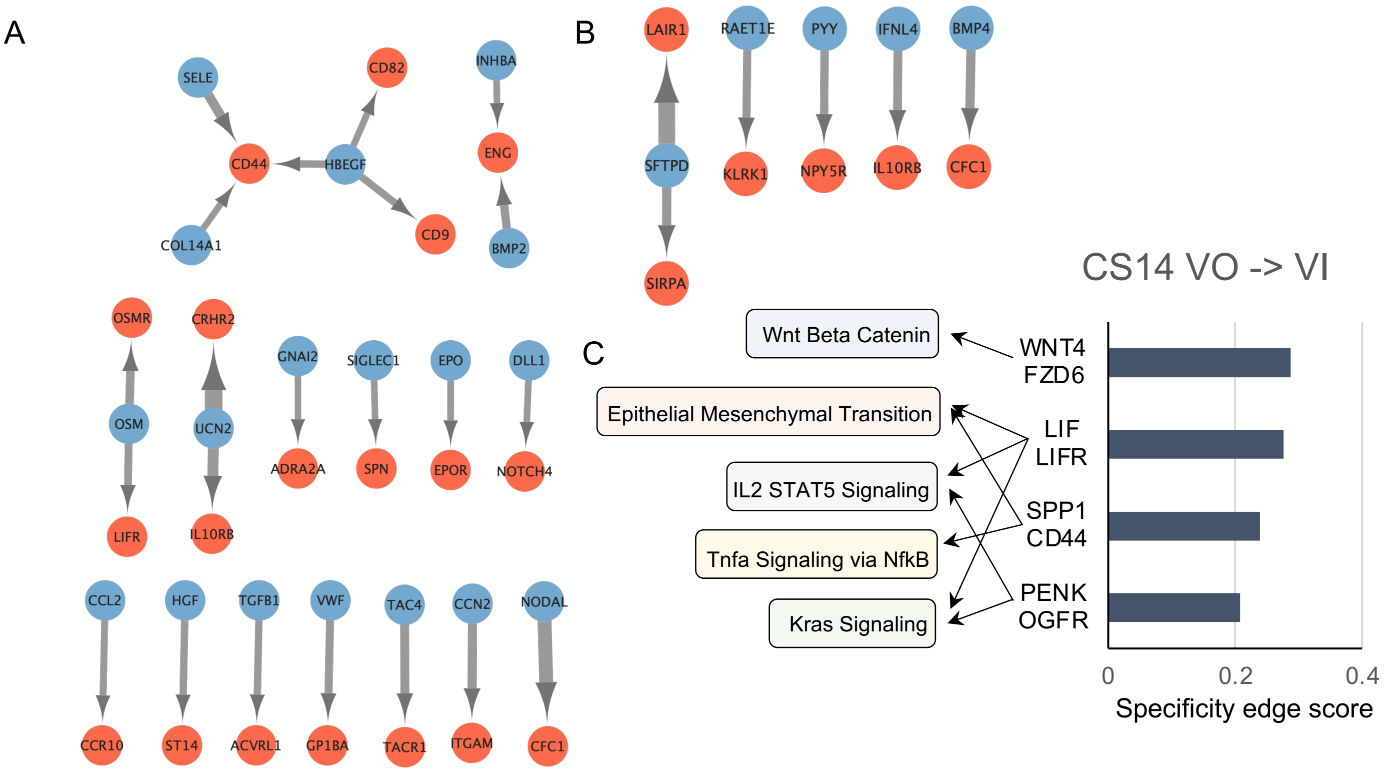

Fig. S4. (related to figure 4). A-B: Network map of top ligand (blue) - receptor (red) interactions for CS13 DI -> VI and CS13 GEP -> VI respectively. Arrow width correlates with specificity score. C: CS14 VO – VI ligand-receptor specificity edge scores for ligands with low molecular weight diffusible proteins (<45kDa) and related pathways.

Development • Supplementary information

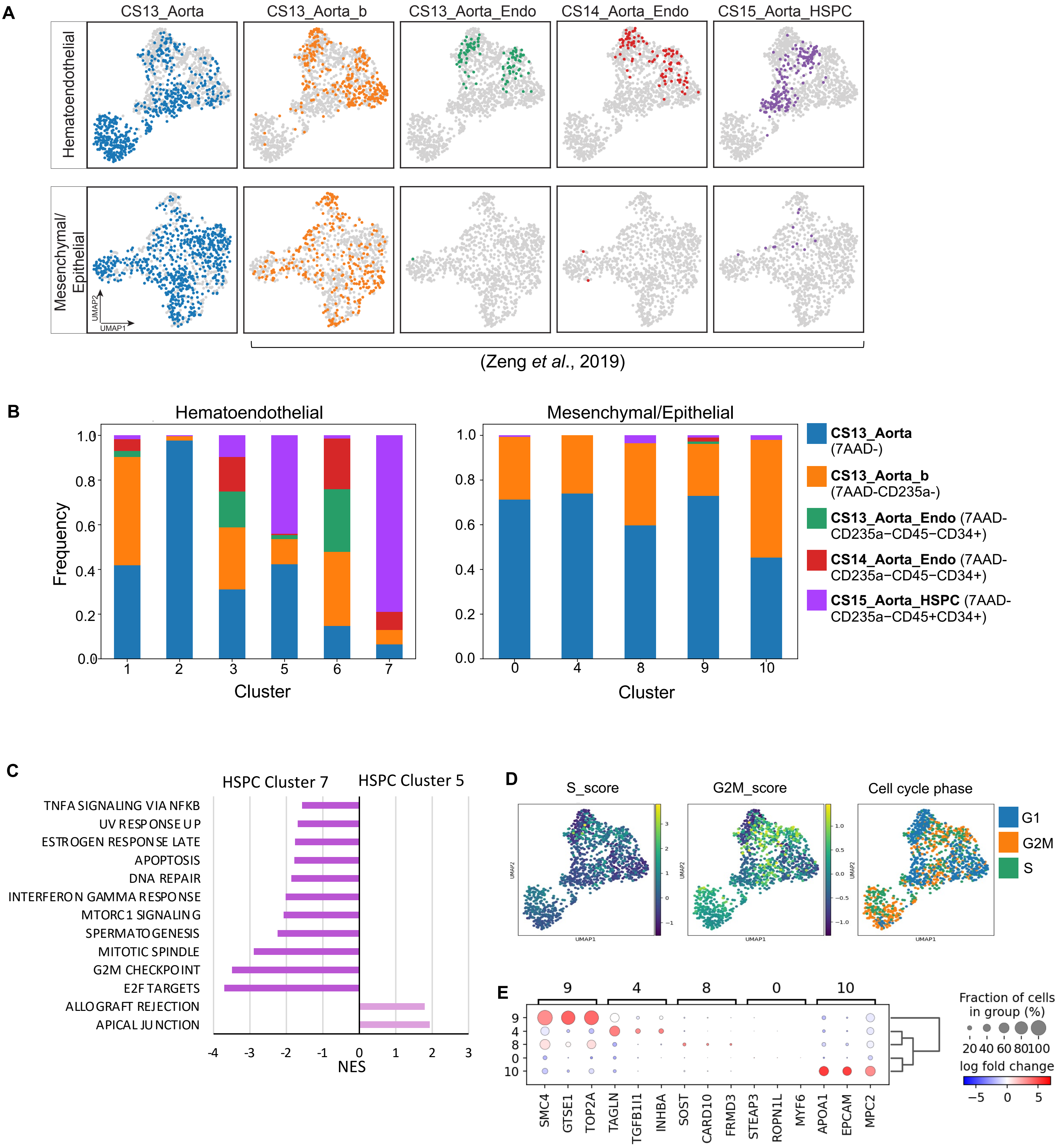

Fig. S5. (related to figure 5): A-E related to haemato-endothelial UMAP in Figure 5A. A: Distribution of cells (Figure 5A) from different stages and cell types from primary data and a previously published dataset (Zeng et al., 2019). B: Frequency of samples within each cluster. C: Differentially enriched pathways for HSPC cluster 5 vs HSPC cluster 7 (FDR<0.1). D: Predicted cell cycle phase. E: Dotplot of top 3 marker genes for mesenchymal/epithelial populations.

Development • Supplementary information

Table S1. Full lists of differentially expressed genes for comparisons described in the manuscripts (CS13 Ventral vs Dorsal, CS14 Ventral vs Dorsal, CS13 vs CS14) and differentially enriched pathways for the same comparisons (GSEA = gene set enrichment analysis). In each comparison title, the first variable corresponds to positive values. BaseMean = mean normalised expression across both conditions.

Available for download at
https://journals.biologists.com/dev/article-lookup/doi/10.1242/dev.201972#supplementary-data

Table S2. Full list of significant genes (p.adj < 0.05) within each grouped dynamic pattern of expression across CS13 – CS17, corresponding to section '**Pathway dynamics in AoV during CS13-CS17'.**

Available for download at
https://journals.biologists.com/dev/article-lookup/doi/10.1242/dev.201972#supplementary-data

Development • Supplementary information
